# Inequities in the utilization of HIV counseling and testing services among undergraduates in mainland China

**DOI:** 10.1186/s12889-021-12252-z

**Published:** 2021-12-04

**Authors:** Jihong Zhan, Guochen Fu, Lei Wu, Mingliang Pan, Yuli Yang, Zhuo Chen, Yu Cao, Yong Li, Hao Wang, Bixiang Wang, Ruyi Du, Yanting Xiong, Wei Liu, Nuo Xu, Xiaobao Xia, Qianqian Li, Fang Ruan, Junfang Wang

**Affiliations:** 1 Department of Preventive Medicine, School of Basic Medical Sciences, Hubei University of Science and Technology, Address: No.88 Xianning Avenue, Xianning City, 437100 Hubei Province China; 2 National Demonstration Center for Experimental General Medicine Education of Hubei University of Science and Technology, Xianning, China

**Keywords:** HCT services, Utilization, Inequities, Undergraduates, China

## Abstract

**Background:**

HIV counseling and testing (HCT) is provided free of charge on college campuses, but very few studies have examined whether HCT uptake is equitably distributed. This cross-sectional study aimed to compare the relative importance of various determinants of HCT uptake among undergraduates in mainland China so as to assess and subsequently to suggest ways to eliminate inequities in its use, guided by the Andersen’s behavioral model.

**Methods:**

A total of 10,665 eligible undergraduates were conveniently selected to complete an online survey. Hierarchical logistic regression analyses were performed to identify the factors associated with HCT utilization.

**Results:**

Only 7.7% of undergraduates had utilized HCT services. HCT uptake was inequitably distributed, since it was mainly determined by predisposing and enabling factors rather than by need factors. Further analysis indicated that HCT uptake was significantly related to two need factors, one enabling factor and six predisposing factors. Those with a higher need [i.e., perceiving themselves to be at higher risk of acquiring HIV infection (AOR = 2.76, 95% CI:2.02–3.78) and engaging in condomless sex (AOR = 1.29, 95% CI:1.00–1.67)] and those with more resources [i.e., being knowledgeable of local AIDS service organization (AOR = 1.59, 95% CI:1.37–1.85)] were more likely to utilize HCT services. Compared to non-heterosexual men, non-heterosexual women (AOR = 0.51, 95% CI:0.37–0.72), heterosexual men (AOR = 0.44, 95% CI:0.33–0.57) and women (AOR = 0.31, 95%CI: 0.24–0.41) were less likely to utilize HCT service. Furthermore, those with more knowledge (AOR = 0.80, 95% CI:0.69–0.94) and taking a positive attitude towards HCT services [i.e, expressing their willingness to utilize HCT services (AOR = 0.68, 95% CI:0.56–0.81) and having recognized the necessity to provide HTC services in the local university (AOR = 0.46, 95% CI:0.36–0.57)] were less likely to utilize HCT services. However, medical students (AOR = 1.34, 95% CI: 1.15–1.56) and non-freshmen (AOR = 1.22, 95% CI:1.03–1.45) were more likely to utilize HCT services.

**Conclusions:**

To increase HCT uptake and simultaneously reduce the remaining inequities, a comprehensive intervention should be continued to target non-heterosexual men and non- freshmen and those with a higher need for HCT services, conduct health education, improve the availability and accessibility of HIV testing services.

**Supplementary Information:**

The online version contains supplementary material available at 10.1186/s12889-021-12252-z.

## Background

Early initiation of sexual activity and subsequent unsafe sexual behaviors (e.g., engaging in condomless sex and having sex with non-regular partners or multiple partners) increased the vulnerability of Chinese young students to HIV infection [1–3]. The figures released by the Chinese Center for Disease Control and Prevention indicated that a total of 3236 young students aged 15–24 years were confirmed to be infected with HIV in 2015, representing an almost 5-fold increase over 2008. Although the newly diagnosed HIV cases have slightly declined over the past few years, more than 3000 cases are reported annually. Male-to-male sexual contact remained as the predominant mode of HIV transmission among young students and accounted for up to 81.8% of new HIV infections in 2017. Furthermore, young students have been recognized as a priority group for HIV prevention measures [4].

HIV counseling and testing (HCT) has been demonstrated to be effective in not only preventing HIV-negatives individuals from becoming infected with the virus by providing HIV prevention education and promoting the adoption of safe behaviors, but also slowing the progression of the disease and controlling symptoms among those already infected by expediting early and accurate diagnosis, timely and effective medical care, and appropriate psycho-social support. More importantly, ensuring that 95% of people living with HIV (PLHIV) know their status is the first and also the most critical step in achieving the UNAIDS 95–95-95 targets by 2030 [5].

In an effort to achieve these goals and to control the national HIV epidemic, the Chinese Center for Disease Control and Prevention has taken several innovative measures such as facilitating HIV self-testing, promoting partner and couples HIV testing with self-test kits and recommending an opt-out approach to screening without routine assessment of risk in healthcare settings. Despite success in some provinces (e.g., Yunnan), China has not reached the 90–90-90 targets set by UNAIDS for 2020 and only 75.7% of people living with HIV were aware of their status at the end of 2019 [5, 6]. It is noted that the utilization rate among college students in China was very low [7–12], ranging from 0.7% [7] to 14.9% [12]. Even among young male students who have sex with men, the HIV testing rate was only 44.3%, based on a meta analysis conducted by Shi et al. [13]. Furthermore, the existing studies indicated that actual uptake and willingness to utilize HCT services was associated with gender [7–9, 11], grade [8, 10], major [7, 11], sexual orientation [7], HIV-related knowledge [7, 10] and stigma [14], having recognized the necessity to provide HTC services in the local university [14], being knowledgeable about free HTC services centers [12, 14], exposure to a peer-led HIV prevention intervention [7], risky sexual behaviors [8, 9], and risk perception [7, 8, 10, 11].

This testing gap, coupled with the importance of early diagnosis and early treatment of HIV infection and the increasing trend of HIV prevalence [6], is driving an interest in promoting HIV testing among young students. Currently, the Chinese health department is delivering free HCT services on college campuses [15], with the goal of increasing the utilization rate of HCT services and ensuring equity of access to HCT services for all undergraduates. Thus, it remains a question whether HCT services are equitably distributed. As described above, previous studies have identified a set of variables associated with HCT uptake among college students in China. However, these associations have been analyzed by only using Person Chi- square test or/and binary logistic regression and no prior research has examined whether inequities in the utilization of HCT services existed among college students in China. Therefore, this study aimed to compare the relative contributions of predisposing, enabling and need factors to HCT utilization among undergraduates in mainland China by using hierarchical logistic regression so as to assess and subsequently to suggest ways to eliminate inequities in its use with the guidance of the Andersen’s Behavioral Model [16–19] which will be discussed in detail below.

### Conceptual framework

The Andersen’s Behavioral Model (ABM) [16–20] has been widely applied in numerous studies to examine the utilization of healthcare services. According to the model, HCT uptake was influenced by three sets of factors. The predisposition of an undergraduate to use HCT services (i.e., predisposing factors) include social- demographic characteristics {e.g., gender [7–9, 11], grade [8, 10], sexual orientation [7] and major [7, 11]} and attitudes or beliefs about medical care, physicians and diseases {e.g., HIV-related knowledge [7, 10] and stigma [14], expressing willingness to utilize HTC service [13] and having recognized the necessity to provide HTC services in the local university [14]}. Factors inhibiting and promoting the utilization of HCT services among college students in China (i.e., enabling factors) can be measured with multiple indicators such as being knowledgeable about free HTC services centers [12, 14], and exposure to a peer-led HIV prevention intervention [7]. Need factors, commonly known as the most immediate cause of HCT uptake, comprise the objective and professional evaluation of need-for HCT services such as early initiation of sexual activity [1] and engaging in risky sexual behaviors [8, 9], and the subjective assessment of need {i.e., risk perception [7, 8, 10, 11]}.

The definition of equity and mutability also need to be addressed here to measure and improve inequities in the utilization of HCT services. The equity dimension is assessed by quantifying the relative contributions of predisposing, enabling and need factors to HCT uptake [16]. More specifically, HCT uptake is considered to be equitably distributed when it is primarily influenced by need factors. Conversely, when HCT uptake is mainly determined by non-need factors (i.e., predisposing or/and enabling factors), inequity of access occurs. Mutability refers to the extent to which a given factor can be altered to influence the distribution of health services. In general, enabling factors and attitudes or beliefs about medical care, physicians and disease are mutable and subject to change, while socio-demographic characteristic and need factors are immutable and cannot be changed in a relatively short period of time.

**Fig. 1:**
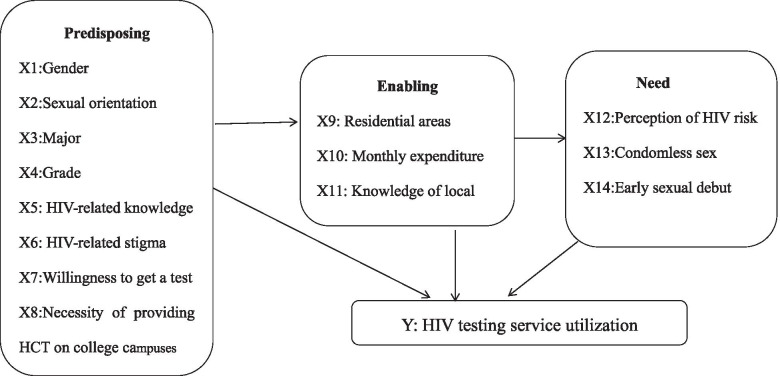
Individual determinants of HCT utilization based on the Andersen's behavioral model

Therefore, guided by the above-mentioned theoretical framework and based on the findings from previous empirical studies, fourteen variables hypothesized to influence HCT utilization were organized into predisposing, enabling and need variables, as indicated in Fig. 1. As a preventive measure of at-risk behavior, HCT utilization is hypothesized to be mainly determined by non-need factors. It is also hypothesized that non-heterosexual men and those with more enabling resources and higher need for HTS services would be more likely to utilize HTS services. The role of other predisposing factors is examined in a more exploratory fashion.

## Methods

### Data collection

A cross-sectional, web-based survey was carried out during a four-month period starting from September 10, 2018. As already described in our recently published paper [19], non-random sampling techniques (i.e., convenience and snowball- sampling) were used to select participants. Due to their convenience and better cooperation, undergraduates currently enrolled in epidemiology, biostatistics, research design and research proposal writing, and evidence-based medicine courses were first organized to fill in the questionnaire in their classrooms during their regularly scheduled class periods in the fifth week of 2018–2019 fall semester. Secondly, a series of incentive measures, including earning extra credits from the Department of Preventive medicine, the honour of Outstanding Volunteer and even a certain amount of money as a reward, have been adopted to stimulate students to invite their friends and acquaintances to participate in the study. All participants were student volunteers enrolled in any of the above-mentioned courses, who were told that they would receive one extra credit point or be paid 0.08 USD for each completed questionnaire and earn up to a total of 50 extra credit points (50 completed questionnaires) worth 5 % of their overall grade. Additionally, completing at least 100 pieces of questionnaires meant that they would also be awarded the Outstanding Volunteer. Meanwhile, the students were specifically informed that they could decline to complete the questionnaire without penalty and those who did not want to participate as a volunteer in this survey were given the option of writing a brief summary of an article, a chapter or a book. In addition, social media platforms such as emails, text messages, Wechat, Sina Weibo and QQ space were utilized to connect with culturally diverse and geographically dispersed undergraduates [19].

This study received ethical clearance from the Research Board of Hubei University of Science and Technology (HUSC). The research team further sought formal permission from the relevant authorities in HUSC. All students involved in the study signed electronic informed consent and were also assured that the questionnaire was anonymous and no identifying information was collected during the survey. More importantly, they were promised that all the data gathered were treated with full confidentiality and were only available to authorized research team members.

### Participants

A total of 21,184 participants currently enrolled as a full-time undergraduate student at HUSC, including 832 third-year medical students enrolled in the above- mentioned four courses, were invited to participate in this survey. The overall response rate was 50.3% (10,665/21184) and the majority (86.9%,723/832) of the sampled medical students participated in the online survey for one extra credit point, after excluding 2085 participants who were not in the age range of 18–25 years, completed the online questionnaire later than January 9, 2019, or not registered as a full-time undergraduate. Indeed, these two figures might overestimate the actual participation rate, because some participants were recruited from other universities. However, we were unable to analyze the characteristics of volunteers and how participants differed from nonparticipants, because all the data were fully anonymized and no identifying information was provided.

Excluding Taiwan, Hong Kong, and Macao, there are 31 provinces in mainland China. The exact number sampled from each province was already presented on the map in our previous work [21]. In general, the eligible 10,665 participants were unevenly distributed across the Chinese mainland (except for Tibet) and were primarily (67.5%) recruited from Hubei province, due to time constrains and the spatial distribution patters of their homes or residences before attending the university.

### Design and content of the questionnaire

Based on the Andersen’s Behavioral model, a self-administrative structured questionnaire (See Supplementary file 1) was developed and validated to collect data about dependent and independent variables.

### The outcome (dependent) variable

The outcome variable for this study was the utilization of HIV counseling and testing services, measured by asking undergraduates to respond to the Yes/No question: “Have you ever utilized HCT services?”

### Explanatory (independent) variables

As demonstrated in Fig. 1, eight predisposing factors included gender, sexual orientation, major, grade, HIV-related knowledge and stigma, willingness to get a test and recognition of the necessity to provide HCT services in the local university. Since male-to-male sexual contact remained the predominant mode of HIV transmission among young students in China, gender and sexual orientation were combined and categorized into four groups (i.e., non-heterosexual men and women, heterosexual men and women) and non-heterosexual men were chosen as the reference group, when performing the multivariate Logistic regression analysis. Consistent with our previous study [14], knowledge was measured by the 12-item scale of Yes/No/I do not know questions, while stigma was based on the Chinese version of Zelaya’s 24-item scale of Yes /No /It depends on the situation statements.

Three enabling factors in this study included residential areas (0 = Rural, 1 = Urban), monthly average expenditure (0 = Low, 1 = High) and awareness of local AIDS service organization. “Together We Fight For Love” (TWFFL) is a volunteer- based organization founded in December 2015, in which students from HUST devote their time to offering free HIV counselling and testing (HCT) in an effort to halt the AIDS epidemic on campus. Consistent with our previous paper [19], awareness of local AIDS service organization was measured by asking the question: “Do you know TWFFL to provide free HCT service?” The undergraduates who answered “Yes” were classified as “aware”, while those who answered “No” were classified as “unaware”.

In this study, self-perceived risk and risky sexual behaviors were respectively used to measure the undergraduate’s subjective and objective need for HCT services. Consistent with our previous study [19], self-perceived HIV risk was assessed on a 5-point scale ranging from 1=“no at all” to 5 = “high”. To increase the overall fitness of the analysis model, students who were unsure about their risk or reported no risk or low risk were classified as low perception, while those self-identified as having moderate or high risk were classified as high perception.

Early initiation of sexual activity [1] and inconsistent condom use [8] were used to reflect actual risk of becoming infected with HIV. Participants were first asked to report whether they had experienced sexual intercourse. Those who answered “Yes” were further asked about their age at first sex and to choose the frequency of condom use (never, once in a while, sometimes and every time) during sexual intercourse. Consistent with our previous study [19], students who started sex before the age of 18 years were classified as early initiators, and inconsistent condom use was defined as failing to use a condom every time when they had sex in the past 6 months.

### Statistical analysis

The statistical analysis of factors associated with HCT uptake was divided into three stages. The first step was to describe the distribution of the dependent and independent variables and compare differences between groups by using two-sided Chi-squared tests. Secondly, a correlation matrix was computed to explore possible associations between independent variables. Additionally, variance inflation factors (VIF) and tolerance values were further calculated to diagnose collinearity in multiple regression. Finally, three sets of independent variables (i.e., predisposing, enabling and need factors) were entered hierarchically into multivariate Logistic regression analysis. Consistent with our previous work [19], only need factors were eligible to be included in the first model. In the second model, enabling factors were added, after adjusting for need factors. Predisposing factors were added to produce the last model, after controlling for both need and enabling factors. The reduction in the − 2 Log likelihood (− 2 LL, a measure of how well the logistic regression model fits with the data, with smaller values indicating a better fit), caused by adding need, enabling or predisposing variables to the Logistic regression model (The degree of freedom is equal to the number of covariates added to the model), were used to evaluate and compare their relative contributions to HCT uptake [19]. Those insignificant independent variables (i.e., Their *p* values were higher than 0.05) were eliminated from the model. The adjusted odds ratios (AOR) and their corresponding 95% confidence intervals (CI) were also calculated to qualify the strength of association between each independent variable and the dependent variable (i.e., HCT uptake). All statistical analyses were performed using IBM SPSS Statistics 25.0.

## Results

### Characteristics of the study participants

**Fig. 2:**
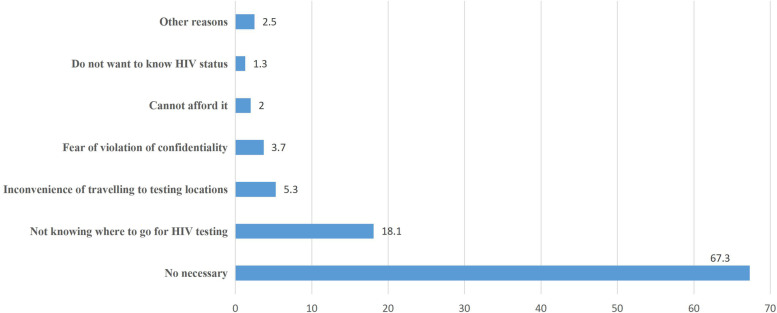
Reasons for not doing HIV Testing among undergraduates in mainland China.

Table 1 presented the characteristics of the study participants. Out of the 10,665 eligible undergraduates, only 818 (7.7, 95% CI:7.2–8.2%) had ever been tested for HIV. Of the remaining 9847 subjects, the most frequently cited reasons for not taking an HIV test were feeling no necessity of being tested (67.3%), not knowing where to get the HIV test (18.1%), inconvenience of travelling to testing locations (5.3%), fear of violation of confidentiality (3.7%), and being unable to afford it (2.0%) (Fig. 2).

**Table 1:** Predisposing, enabling and need characteristics of the 10665 undergraduates in mainland China (n=10665)

A		Total (n=10665)		Ever (n=818)		Never (n=9847)		χ2	P
		n	%	n	%	n	%		
**Predisposing factors**									
X1: Gender									
	0=Female	6137	57.5	388	47.4	5749	58.4	37.07	<0.001
	1=Male	4528	42.5	430	52.6	4098	41.6		
X2:Sexual orientation									
	0=Non-heterosexuals	1231	11.5	166	20.3	1065	10.8	66.45	<0.001
	1=Heterosexuals	9434	88.5	652	79.7	8782	89.2		
X3: Major									
	0=Non-Medical	7472	70.1	514	62.8	6958	70.7	22.05	<0.001
	1=Medical	3193	29.9	304	37.2	2889	29.3		
X4: Grade									
	0=Freshmen	3008	28.2	198	24.2	2810	28.5	7.00	0.008
	1=Non-freshmen	7657	71.8	620	75.8	7037	71.5		
X5: HIV-related knowledge									
	0=Low	4084	38.3	379	46.3	3705	37.6	24.23	<0.001
	1=High	6581	61.7	439	53.7	6142	62.4		
X6: HIV-related stigma									
	0=High	5752	53.9	492	60.1	5260	53.4	13.77	<0.001
	1=Low	4913	46.1	326	39.9	4587	46.6		
X7: Willingness to utilize HTC service									
	0=No	1774	16.6	208	25.4	1566	15.9	49.41	<0.001
	1=Yes	8891	83.4	610	74.6	8281	84.1		
X8: Recognition of the necessity to provide HCT in the local university									
	0=No	706	6.6	133	16.3	573	5.8	133.17	<0.001
	1=Yes	9959	93.4	685	83.7	9274	94.2		
**Enabling factors**									
X9: Residential areas									
	0=Rural	7207	67.6	525	64.2	6682	67.9	4.66	0.031
	1=Urban	3458	32.4	293	35.8	3165	32.1		
X10: Monthly expenditure									
	0=Low	9746	91.4	739	90.3	9007	91.5	1.22	0.270
	1=High	919	8.6	79	9.7	840	8.5		
X11: Knowledge of local AIDS service organization									
	0=No	5276	49.5	337	41.2	4939	50.2	24.25	<0.001
	1=Yes	5389	50.5	481	58.8	4908	49.8		
**Need factors**									
X12: Perception of HIV risk									
	0=Low	10403	97.5	754	92.2	9649	98.0	106.51	<0.001
	1=High	262	2.5	64	7.8	198	2.0		
X13: Condomless sex									
	0= No	9870	92.5	723	88.4	9147	92.9	22.22	<0.001
	1=Yes	795	7.5	95	11.6	700	7.1		
X14: Early sexual debut									
	0= No	10046	94.2	738	90.2	9308	94.5	25.62	<0.001
	1=Yes	619	5.8	80	9.8	539	5.5

An analysis of predisposing factors indicated that 42.5% were male and 88.5% self-identified as heterosexuals. About seven tenths of them pursued non-medical degrees (70.1%), completed more than 1 year of college study (71.8%). Approximately one half of students demonstrated a low level of knowledge of HIV transmission (38.3%) and stigmatizing attitudes towards PLHIV (53.9%). However, a vast majority of respondents expressed willingness to utilize HTC services (83.4%) and recognized the necessity to provide HTC services in the local university (93.4%).

With respect to enabling variables, more than two-thirds of undergraduates resided in rural areas (67.6%) and less than one-tenth (8.6%) reported that their monthly expenditure on food consumption, clothing and entertainment was higher than 2000 Yuan RMB [1Yuan RMB = 0.1493US dollars (October, 2020 rate)]. Furthermore, only one-half (50.5%) knew about local AIDS service organization.

The need variables suggested that undergraduates tended to systematically underestimate their risk relative to their self-reported exposure to unsafe sex, because only a tiny minority (2.5%) perceived themselves to be at high risk of acquiring HIV infection, yet 7.5% reported that they had in fact engaged in condomless sex. And even 5.8% of respondents were classified as early sexual initiators.

### Correlations between independent variables and dependent variable

As indicated in the last column in Table 1, all other independent variables (except for monthly expenditure) had statistically significant associations with HCT uptake. When compared with those who had never been tested, undergraduates reporting a previous HIV test were significantly more likely to be male, non- heterosexuals and non-freshmen, pursue a medical degree, reside in rural areas, have a lower level of basic HIV transmission knowledge and a stigmatizing attitude towards PLHIV, express their unwillingness to utilize HCT services, have not recognized the necessity to provide HTC services in the local university, be knowledgeable of local AIDS service organization, perceive themselves to be at high risk of acquiring HIV, engage in condomless sex and start having sex at an earlier age.

### Multicollinearity diagnosis

As indicated in Table 2, there were statistically significant but weak correlations between some independent variables. Furthermore, tolerance (ranging 0.65 between 0.98) and VIF values (defined as the reciprocal of tolerance, ranging 1.02 between 1.54) (See Table 3) were all within acceptable limits (i.e., tolerance values were all greater than 0.10 and VIF values were lower than 10), indicating no evidence of multicollinearity [22]. Therefore, no variable was excluded from further analysis.

**Table 2:** The matrix of Pearson correlation coefficients of factors associated with HIV testing (Y)

Variables	X1	X2	X3	X4	X5	X6	X7	X8	X9	X10	X11	X12	X13	X14
X1:Gender	-													
X2:Sexual orientation	-0.59^***^	-												
X3:Major	-0.01	-0.02	-											
X4:Grade	-0.08^***^	0.03^***^	0.04^***^	-										
X5: HIV-related knowledge	0.02	0.03^**^	0.11^***^	0.02	-									
X6: HIV-related stigma	-0.05^***^	0.04^***^	0.07^***^	-0.06^***^	0.25^***^	-								
X7: Willingness to utilize HTC service	-0.06^***^	0.05^***^	0.03^***^	-0.02	0.10^***^	0.10^***^	-							
X8: Recognition of the necessity to provide HTC	-0.06^***^	0.08^***^	-0.01	-0.02	0.15^***^	0.10^***^	0.23^***^	-						
X9: Residential areas	0.05^***^	-0.06^***^	0.01	0.00	0.01	-0.01	0.01	-0.01	-					
X10: Monthly expenditure	0.03^***^	-0.04^***^	-0.04^***^	0.02	-0.01	-0.04^***^	0.00	-0.01	0.18^***^	-				
X11: Knowledge of local AIDS service organization	0.02^*^	0.01	0.15^***^	0.02	0.10^***^	0.06^***^	0.09^***^	0.06^***^	0.02	-0.03	-			
X12: Perception of HIV risk	0.03^***^	-0.06^***^	0.01	0.03	-0.08^***^	-0.09^***^	-0.07^***^	-0.13^***^	-0.01	0.00	-0.04^***^	-		
X13: Condomless sex	0.08^***^	-0.10^***^	-0.01	0.04^***^	-0.04^***^	-0.06^***^	0.00	-0.04^***^	0.05^***^	0.10^***^	-0.05^***^	0.05^***^	-	
X14: Early sexual debut	0.11^***^	-0.13^***^	0.01	-0.01	-0.05^***^	-0.08^***^	0.02	-0.04^***^	0.07^***^	0.09^***^	0.02	0.03^***^	0.34^***^	-

^*^P ≤ 0.05, ^**^P ≤ 0.01, and ^***^P ≤ 0.001

**Table 3:** Collinearity diagnosis of factors associated with HIV testing

Variables	Tolerance	VIF
X1:Gender	0.65	1.54
X2:Sexual orientation	0.65	1.54
X3:Major	0.96	1.04
X4:Grade	0.98	1.02
X5: HIV-related knowledge	0.90	1.11
X6: HIV-related stigma	0.91	1.10
X7: Willingness to utilize HTC service	0.93	1.08
X8: Recognition of the necessity to provide HTC	0.91	1.10
X9: Residential areas	0.96	1.04
X10: Monthly expenditure	0.95	1.05
X11: Knowledge of local AIDS service organization	0.96	1.04
X12: Perception of HIV risk	0.97	1.03
X13: Condomless sex	0.87	1.15
X14: Early sexual debut	0.86	1.16

### Hierarchical logistic regression analysis

Table 4 showed the results of hierarchical logistic regression analyses of need, enabling and predisposing variables on HTC uptake. Model 1 only included three need factors and indicated that all the three measures of need (i.e., perception of HIV risk, condomless sex and early sexual debut) were significantly associated with HCT uptake. The initial − 2 Log likelihood (− 2LL) was 5772.62 for Model 0 (a model with no independent variables) and this model was used as a basis to evaluate a decrease in − 2 LL. The model with three need factors alone (Model 1) had a − 2 LL of 5675.28 and the difference between the − 2LL (5772.62) for Model 0 and the -2 LL (5675.28) for Model 1 was Block Chi-square (97.34) which was significant at *p* < 0.001. In other words, adding three need factors to the model (Model 1) induced a significant reduction in − 2 LL (χ2 = 97.34; df = 3; *P* < 0.001). When two enabling factors were added to the model (Model 2), knowledge of local AIDS service organization but not residential area was statistically significant. Adding enabling factors to the model with need factors further contributed to the reduction in the value of − 2 LL and thus improved the model’s performance (χ2 = 33.11; df = 2; P < 0.001). When predisposing factors were also added (Model 3), all other predisposing variables except for stigma towards PLHIV were still statistically significant, while early sexual debut lost its statistical significance. A comparison of Model 2 and Model 3 indicated that the value of − 2 LL of Model 3 was significantly decreased (χ2 = 196.15; df = 9; P < 0.001) and Model 3 was preferred over Model 2. Therefore, the final model, Model 3, had the best fit with the data. Furthermore, predisposing and enabling factors produced more than twice the reduction in − 2 LL and thus exerted much stronger effects than need factors, indicating the existence of inequities in the utilization of HCT services.

**Table 4:** Logistic regression analysis of factors associated with HIV testing (*N*=10665)

Variables	Model 1			Model 2			Model 3		
	P value AOR	AOR	95% CI	P value AOR	AOR	95% CI	P value AOR	AOR	95% CI
**Block 1: Need factors**									
X12: Perception of HIV risk (0=Low, 1=High)	<0.001	3.95	2.95-5.30	<0.001	4.21	3.13-5.65	<0.001	2.76	2.02-3.78
X13: Condomless sex (0=No, 1=Yes)	0.010	1.39	1.08-1.79	0.005	1.44	1.12-1.85	0.048	1.29	1.00-1.67
X14: Early sexual debut (0=No, 1=Yes)	0.001	1.59	1.21-2.08	0.002	1.53	1.16-2.00	0.059	1.31	0.99-1.73
**Block 2:Enabling factors**									
X9: Residential areas (0=Rural, 1=Urban)				0.084	1.14	0.98-1.33	0.199	1.11	0.95-1.29
X11: Knowledge of local AIDS service organization (0=No, 1=Yes)				0.000	1.50	1.30-1.74	<0.001	1.59	1.37-1.85
**Block 3: Predisposing factors**									
X2: Sexual orientation * (Ref:Non-heterosexual men)									
Non-heterosexual women							<0.001	0.51	0.37-0.72
Heterosexual men							<0.001	0.44	0.33-0.57
Heterosexual women							<0.001	0.31	0.24-0.41
X3: Major (0=Non-Medical,1=Medical)							<0.001	1.34	1.15-1.56
X4: Grade (0=Freshmen, 1=Non-freshmen)							0.022	1.22	1.03-1.45
X5: HIV-related knowledge (0=Low, 1=High)							0.007	0.80	0.69-0.94
X6: HIV-related stigma (0=High, 1=Low)							0.413	0.94	0.80-1.10
X7: Willingness to utilize HTC service (0=No, 1=Yes)							<0.001	0.68	0.56-0.81
X8: Necessity of providing HCT (0=No, 1=Yes)							<0.001	0.46	0.36-0.57
-2 Log likelihood	5675.28			5642.17			5446.02		
Change of −2 log likelihood (χ2), d.f.	97.34,3			33.11,2			196.15,9		
P value	<0.001			<0.001			<0.001		

*d.f.* degree of freedom; *OR* odds ratio; *CI* confidence interval

In the final model (Model 3), two need factors, one enabling factor and six predisposing factors had statistically significant associations with HCT uptake. Those with a higher need for HCT services [i.e., perceiving themselves to be at higher risk of acquiring HIV infection (AOR = 2.76, 95% CI:2.02–3.78) and engaging in condomless sex (AOR = 1.29, 95% CI:1.00–1.67)] and those with more enabling resources [i.e., being knowledgeable of local AIDS service organization (AOR = 1.59, 95% CI:1.37–1.85)] were more likely to utilize HCT services. Compared to non-heterosexual men, non-heterosexual women (AOR = 0.51, 95% CI: 0.37–0.72), heterosexual men (AOR = 0.44, 95% CI:0.33–0.57) and women (AOR = 0.31, 95%CI: 0.24–0.41) were less likely to utilize HCT services. Furthermore, those with more knowledge (AOR = 0.80, 95% CI:0.69–0.94) and taking a positive attitude towards HCT services [i.e, expressing their willingness to utilize HCT services (AOR = 0.68, 95% CI:0.56–0.81) and having recognized the necessity to provide HTC services in the local university (AOR = 0.46, 95% CI:0.36–0.57)] were less likely to utilize HCT services. However, medical students (AOR = 1.34, 95% CI: 1.15–1.56) and non-freshmen (AOR = 1.22, 95% CI: 1.03–1.45) were more likely to utilize HCT services.

## Discussion

### Main findings of this study

In this cross-selectional study, the overall utilization rate of HCT services was 7.7%, which was higher than the rates reported by other authors [7–11], but lower than the level (14.9%) calculated by Qin, Gao, Zhu, Zhang, Kong and Chen [12]. Similar to previous studies’ findings, a vast majority of students expressed their willingness to use HCT services [7, 14] and felt the necessity of providing HCT services on college campuses [14]. However, feeling no necessity of being tested (67.3%) and not knowing where to get an HIV test (18.1%) were cited as major reasons for non- participation. Furthermore, our study also added to the growing body of evidence that the discordance existed between self-perceived and reported HIV risk [23–25]. Based on the analysis of Pringle et al. (2013), this discordance might be caused by optimistic bias (the belief that negative events are less likely to befall on oneself than others), denial and distancing (refusal to accept an unpleasant truth), and downward comparison (when a person compares oneself to those less fortunate) [23]. This underscores the urgent need for promoting self-realization of HIV risk [8, 10, 24] when launching a large-scale testing campaign among college student in order to achieve the ambitious “95–95-95″ targets by 2030 [5] and the final goal of three Zeros.

Based on the Andersen’s behavioral model, this study revealed that HCT uptake was inequitably distributed, since it was mainly influenced by predisposing and enabling factors rather than need factors of undergraduates. More specifically, HCT uptake was significantly related to six predisposing factors (sexual orientation, major, grade, HIV-related knowledge, willingness to utilize HTC services and recognition of the necessity to provide HCT), one enabling factors (knowledge of local AIDS service organization) and two need factors (condomless sex and perception of HIV risk).

The effects of sexual orientation were consistent with a meta analysis conducted by Shi et al. in 2018 in which non-heterosexuals men were more likely to utilize HCT services [13]. This phenomenon could be explained by the following two factors. First, previous HIV educational campaigns focused primarily on individuals at high risk for HIV infection such as men who have sex with men and sex workers, which might perpetuate the belief that participants’ social identities, rather than their risky sexual behaviors were closely correlated with HIV risk. Second, condomless anal intercourse is considered the highest-risk sexual behavior and male-male sexual contact remains the predominant mode of HIV transmission among Chinese young students.

Older students (i.e., non-freshmen) were found to be more likely to utilize HCT services than younger individuals, possibly due to the fact that the chances of being exposed to HIV infection and subsequent risk perception increases with age. As with our previous study [14], medical students and those who knew about free HIV testing centers were more likely to utilize HCT services. These findings support the idea that exposure to accurate levels of HIV and AIDS information through the media, health education and access to HCT services may improve the knowledge about risks for HIV infection and dispel the stigma associated with HIV infection, which can help to a certain extent to reduce optimistic bias and finally contribute to accurate risk perception and actual use of HCT services.

Compared to their respective counterparts, students with more knowledge of HIV transmission and those who expressed their willingness to use HCT services and felt the necessity of providing HCT services on college campuses were found to be less likely to utilize HCT services. This finding is not surprising as it fits knowledge- attitude-belief-practice model. Meanwhile, our data also indicated that those with higher levels of knowledge were more likely to have positive attitudes and practices towards preventive health measures, and were consequently less likely to engage in risky sexual activities (See Table 2), and certainly less likely to use HCT services.

The emergence of condomless sex and self-perceived risk of HIV infection as having a strong positive association with utilization of HCT services was consistent with previous findings in which those who perceived themselves to be at risk of HIV infection [8] and engaged in risky sexual behaviors [8] were more likely to utilize the services than their counterparts. This finding supports the notion that undergraduates with higher risk perception and those who had engaged in condomless sex are more likely to require and consequently utilize HCT services.

### Limitation

The first limitation of this study is the cross-sectional design, which limits its ability to confirm causal relationships. Thus, more studies need to be conducted based on a prospective and longitudinal design. Second, a combination of convenience and snowball sampling was applied to recruit students mainly from HUST and thus our sample was not fully representative. It was therefore difficult to generalize the findings to undergraduates from other universities and colleges in mainland China. Third, this study relied mainly on self-reported measurements and might be prone to social desirability bias due to the sensitivity of sexual topics. However, this type of bias might be minimized via anonymous online survey. Fourth, according to the Chinese nominal age system, a person is counted as 1 year old instead of zero year old on the day of his birth and becomes 1 year older each year on the day when the Chinese New Year is celebrated. Thus, he would be 2 years old at the turn of the Chinese New Year, if his birth happens on the last day of the Lunar Year. In other words, the nominal age is usually exaggerated by one to 2 years as compared with the actual age. When asked about their age, some students might report their nominal age instead of their actual age, thus resulting in inaccurate statistics. Given the fact the majority of students come to HUST to pursue a three-, four- or five-year degree at around the age of 18 years, which means that their ages range between 18 and 23 years. However, some university students might upgrade from junior college students or enroll later than the compulsory school attendance, the age range in this study was thus widened to be 18–25 years. Therefore, the respondents in this study were simply asked to select their age from one of four categories (younger than 18 years old = 1; 18–25 years old = 2; 26–29 years old = 3; and 30 years or older = 4) to determine whether they meet our eligibility criteria to be considered for such an analysis. In addition, because Chinese university students in the same grade almost fall into the same category, age in this study was crudely estimated with the students’ grade. Fifth, with the wide use of mobile phones and internet, the web-based survey was easier to participate, especially when the students were allowed to complete during regular class time. Therefore, the participation rate (86.9%) in this study was higher that (75%) reported by Elicker et al. (2010) [26]. However, due to the lack of intrinsic motivation and weak extrinsic incentives such as failure to require participation, low values of extra credit or cash incentives [26], some students did not participate at all. In order to improve the representativeness of research samples and the quality of the survey research, greater effort should be made to increase students’ interest and enthusiasm by communicating the intrinsic value of scientific research and research participation. Finally, some factors potentially associated with HCT uptake, including individual behaviours such as drug use [7, 8] and having an STD history, HIV status of sexual partners, college characteristics (e.g., vocation school or college) [8], were not investigated in the present study and merited further research.

### Implications of the study

In spite of the above-mentioned limitations, the findings from our study have several implications for the design and implementation of HIV testing programs on college campuses. To the best of our knowledge, this is the first publication that employs the Andersen’s behavioral model as a theoretical framework, together with hierarchical Logistic regression model, to examine equitable distribution of HCT services among a large and diverse undergraduate sample. Our findings suggested that HCT utilization was inequitably distributed and participants who had ever utilized HCT services were mainly those with a higher need for HCT services (i.e., engaging in condomless sex and perceived themselves to be at high risk of acquiring HIV infection) and those with more enabling resources (i.e., being knowledgeable of local AIDS service organization). Furthermore, non-heterosexual men, medical students and non-freshmen and those with lower knowledge of HIV and taking a negative attitude towards HCT services (i.e, expressing unwillingness to utilize HTC service and feeling no necessity of providing HCT on college campuses) were also found to be more likely to use HCT services. In order to increase the utilization rate of HCT services and simultaneously reduce the remaining inequities, three main types of intervention are recommended.

First, target non-heterosexual men, non-freshmen and those with a higher need for HCT services. The tendency for non-heterosexual men, non-freshmen and those with a higher need (i.e., engaging in condomless sex and perceiving themselves to be at higher risk of acquiring HIV) to utilize HCT services would be labeled as equitable and immutable. Therefore, free routine opt-out HIV testing should be immediately implemented among students exhibiting these characteristics [27], while our long- term goals should be set to recommend such testing option to all undergraduates.

Second, conduct health education: Health education should be conducted to improve undergraduates’ knowledge about HIV transmission and prevention, enhance their willingness to utilize HCT services and raise their awareness of local AIDS service organization. Furthermore, it should be emphasized that HIV risk is dependent on exposure to risky behaviors such as the sharing of HIV-contaminated needles and unprotected sex, rather than participants’ social identities.

Third, improve the availability and accessibility of HIV testing services. Our results indicated that a vast majority of undergraduates expressed willingness to utilize HTC services (83.4%) and recognized the necessity to provide HTC services in the local university (93.4%), and not knowing where to get an HIV test was identified as one of the key barriers for undergraduates to utilization of HTC services. Therefore, continued effort should be needed to recruit and train peer volunteers to provide free HCT services in college campus. However, due to the existence of heterogeneous HIV testing preferences [28–30], a variety of HIV testing options should be provided to achieve the ambitious “95–95-95” targets by 2030 [5] and the final goal of three Zeros.

## Conclusion

Our findings suggested that equal use of HCT services for equal needs had not been achieved, and that HCT uptake were significantly related to six predisposing characteristics, one enabling variable and two need factors. Three types of measures such as targeting non-heterosexual men and non-freshmen and those with a higher need for HCT services, conducting health education, improving the availability and accessibility of HIV testing services are therefore recommended to increase HCT uptake and simultaneously reduce the remaining inequities.

## Supplementary Information

**Additional file 1.**

## Data Availability

The data set supporting the results of this article is available in the Harvard Dataverse repository at: https://dataverse.harvard.edu/dataset.xhtml?persistentId=doi:10.7910/DVN/GBFCTK.

## Abbreviations

AOR: Adjusted odds Ratio
CI: Confidence Interval
HIV: Human Immunodeficiency Virus
HCT: HIV counseling and testing
HUST: Hubei University of Science and Technology
PLHIV: People living with HIV

## References

[1] Ma Q, Ono-Kihara M, Cong L, Xu G, Pan X, Zamani S (2009). Early initiation of sexual activity: a risk factor for sexually transmitted diseases, HIV infection, and unwanted pregnancy among university students in China. BMC Public Health.

[2] Li J, Li S, Yan H, Xu D, Xiao H, Cao Y (2015). Early sex initiation and subsequent unsafe sexual behaviors and sex-related risks among female undergraduates in Wuhan. China APJPH.

[3] Ge L, Cui Y, Li D, Li P, Guo W (2015). Cross-sectional study on AIDS/HIV related sexual behavior among students from 2010-2015. Chin J Sch Health.

[4] Lv F (2017). Key strategy of the China action plan for the thirteen five-year plan for combating and prevention of AIDS. Chin J Prev Med.

[5] UNAIDS. Understanding Fast-Track: accelerating action to end the AIDS epidemic by. 2030:2015.

[6] Chuai Z, Zhang Y, Zhao Y, Yan J, Sun J, Wang Y (2020). Latest AIDS epidemic in global and China. Infect Dis Info.

[7] Chen M, Chen W, Xu H, Cai Y, Zhong F, Chen X (2018). Analysis of factors associated with willingness to accept peer HIV voluntary counseling and testing among college students in Guangzhou. Chin J Sch Health..

[8] Ding L, Lin P, Li Y, Lin Z, Fu X, Long Q (2017). HIV testing and associated factors among young students in Guangzhou. Chin J AIDS STD..

[9] Wen M, Zhu G, Sun X, Liu Y (2015). Intent to accept the HIV antibody testing and its need assessment. Chin J Sch Health..

[10] Jiang J, Pan X, Yang J, Ma Q, Chen L, He L (2016). Willingness for HIV test and associated factors among 535 college students who had sex in Zhejiang province. Chin J Epidemipol.

[11] Liang H, Liang X, Guo Y, Chen S, Xie N, He S (2020). Willingness to accept HIV/AIDS voluntary counseling and testing service and its influencing factors among young students in China, 2018. Pract Prev Med.

[12] Qin Q, Gao Y, Zhu H, Zhang Z, Kong J, Chen B (2017). Investigation on cognition and willingness of HIV test among college students in Ma'anshan City. Occup and Health.

[13] Shi A, Zhang Z, Wang J, Zhu X, Zhao Y, Wang W, & Zhang H. Meta analysis of the high risky behaviors and detection rate of HIV infection among MSM students in Mainland China. Chin J Sch Health.2018;39(5):702-.

[14] Fu G, Shi Y, Yan Y, Li Y, Han J, Li G (2018). The prevalence of and factors associated with willingness to utilize HTC service among college students in China. BMC Public Health.

[15] Du Y, Xu H (2020). Utilization of HIV testing and counseling services and its influencing factors among young students in China. Chin J AIDS STD.

[16] Andersen R, Aday LA (1978). Access to medical care in the U.S.: realized and potential. Med Care.

[17] Kempen G, Suurmeijer T (1991). Professional home care for the elderly: an application of the Andersen-Newman model in the Netherlands. Soc Sci Med.

[18] Andersen R, Newman JF (1973). Societal and individual determinants of medical care utilization in the United States. Milbank Q.

[19] Ruan F, Fu G, Yan Y, Li Y, Shi Y, Luo L (2019). Inequities in consistent condom use among sexually experienced undergraduates in mainland China: implications for planning interventions. BMC Public Health.

[20] Nelson CI, Wright CD, Brumbaugh JT, Neiswanger K, Crout RJ, Lilly CL, et al. Predictors of use of dental care by children in north-central Appalachia in the USA. PLOS ONE.2021:16(7): e0250488. 10.1371/journal.pone.0250488 PMC8297786 34292949

[21] Ruan F, Fu G, Zhou M, Luo L, Chen J, Hua W (2019). Application of the Chinese version of Zelaya's HIV-related stigma scale to undergraduates in mainland China. BMC Public Health.

[22] Wang J, Wu Y, Zhou B, Zhang S, Zheng W, Chen K (2010). Factors associated with non-use of inpatient hospital care service by elderly people in China. HEALTH SOC CARE COMM.

[23] Pringle K, Merchant RC, & Clark MA . Is self-perceived HIV risk congruent with reported HIV risk among traditionally lower HIV risk and prevalence adult emergency department patients? Implications for HIV testing. AIDS Patient Care and STDs.2013;27(10):573–84. 10.1089/apc.2013.0013 PMC3837562 24093811

[24] Alexovitz KA, Merchant RC, Clark MA, Liu T, Rosenberger JG, Bauermeister J (2018). Discordance of voluntary HIV testing with HIV sexual risk-taking and self-perceived HIV infection risk among social media-using black, Hispanic, and white young-men-who-have-sex -with-men (YMSM). AIDS Care.

[25] Oostrom L, Rosentel K, Motley D, Hill BJ (2020). Discordance in objective and self-perceived HIV risk: a potential barrier to pre-exposure prophylaxis in young gay and bisexual men. J Assoc Nurses AIDS Care.

[26] Elicker JD, McConnell NL, Hall RJ (2010). Research participation for course credit in introductory psychology: why don't people participate?. Teach Psychol.

[27] Niu L, Wang Z, Fang Y, Ip M, Lau JTF (2019). Behavior intention to use routine opt-out HIV testing in primary care settings among men who have sex with men in China. AIDS Care.

[28] Nwaozuru U, Lwelunmor J, Salah S, Ezechi O, Obiezu-Umeh C, Tucker JD (2019). Preferences for HIV testing services among young people in Nigeria. BMC Health Serv Res.

[29] Frye V, Wilton L, Hirshfield S, Chiasson MA, Lucy D, Usher D (2018). Preferences for HIV test characteristics among young, black men who have sex with men (MSM) and transgender women: implications for consistent HIV testing. PLoS One.

[30] Ostermann J, Njau B, Brown DS, Mühlbacher A, Thielman N (2014). Heterogeneous HIV testing preferences in an urban setting in Tanzania: results from a discrete choice experiment. PLoS One.

