# Supplementary material for: Inequities in the utilization of HIV counseling and testing services among undergraduates in mainland China
Source: BMC Public Health. 2021 Dec 4;21:2216. doi: 10.1186/s12889-021-12252-z (PMC8645148; doi:10.1186/s12889-021-12252-z)
Supplement: Supplementary file 1 — Additional file 1. [file 12889_2021_12252_MOESM1_ESM.docx]

**Supplementary file 1: The structured questionnaire**

**Part I: Sociodemographic Characteristics**

1. What's your gender? ① Male ② Female
2. How old are you?
3. Under the age of 18 years old ②18-25 ③26-29 ④ No less than 30 years old
4. Where do you live mostly before going to college?
5. City  ②District/County/Town ③ Countryside
6. What is your major?

① Preventive medicine ②Clinical medicine ③ Psychology ④ Social Science

⑤ Other (Please specify)

1. What is your grade?
2. Freshmen ② Sophomore ③ Junior ④ Senior ⑤ Graduate and above
3. What is your average monthly expenditure in Yuan RMB?
4. Less than 500 ②500-999 ③ 1000-1999 ④ More than 2000
5. What is you sexual orientation?
6. Heterosexual ② Homosexual ③ Bisexual ④ Not sure

**Part II: HIV-related knowledge**

1. Can mosquito bites transmit HIV?

① Yes ② No ③ I do not know

1. Can a person be diagnosed with HIV by his appearance?

① Yes ② No ③ I do not know

1. Can causal contact with patients transmit HIV?

① Yes ② No ③ I do not know

1. Is HIV transmitted from an infected mother to her baby?

① Yes ② No ③ I do not know

1. Can receiving blood and blood products lead to HIV infection?

① Yes ② No ③ I do not know

1. Can correct and consistent use of condoms reduce the risk of HIV transmission?

① Yes ② No ③ I do not know

1. Is HIV a highly contagious, incurable disease?

① Yes ② No ③ I do not know

1. At present, is HIV spreading rapidly among young students in China, transmitted primarily through male homosexual contact and secondly through heterosexual contact? ① Yes ② No ③ I do not know
2. After taking high-risk behaviors such as needle-sharing among drug users and unprotected sexual contact, should a person immediately seek HIV voluntary counseling and test? ① Yes ② No ③ I do not know
3. Can having only one uninfected sex partner reduce the risk of HIV transmission?

① Yes ② No ③ I do not know

1. Have the rights of PLHIV been legally protected, including the rights of marriage, employment,education and medical care?

① Yes ② No ③ I do not know

1. Can use of new type of drugs such as methamphetamine increase the risk of transmitting HIV? ① Yes ② No ③ I do not know

**Part III: HIV-related stigma ( PLHIV: People living with HIV)**

1. Do you agree with the statement "You could become infected with HIV if you are kissing PLHIV"? ①Yes ② No ③ It depends on the situation statements
2. Do you agree with the statement " You could become infected with HIV if you are exposed to cough or sneeze of PLHIV "?

①Yes ② No ③ It depends on the situation statements

1. Do you agree with the statement " You could become infected with HIV if you are exposed to the saliva of PLHIV "?

①Yes ② No ③ It depends on the situation statements

1. Do you agree with the statement "You could become infected with HIV if you are exposed to the sweat of PLHIV "? ①Yes ② No ③ It depends on the situation statements
2. Do you agree with the statement "You could become infected with HIV if you are exposed to the urine of PLHIV"? ①Yes ② No ③ It depends on the situation statements
3. Do you agree with the statement "You could become infected with HIV if you are playing with PLHIV "? ①Yes ② No ③ It depends on the situation statements
4. Do you agree with the statement "HIV is punishment for bad behavior "?

①Yes ② No ③ It depends on the situation statements

1. Do you agree with the statement "It is women prostitutes who spread HIV "?

①Yes ② No ③ It depends on the situation statements

1. Do you agree with the statement "PLHIV are promiscuous "?

①Yes ② No ③ It depends on the situation statements

1. Do you agree with the statement "Only PLHIV caused by blood transfusion should be cared for and treated "? ①Yes ② No ③ It depends on the situation statements
2. Do you agree with the statement "Youths might be badly influenced by PLHIV and participate in illegal activities "? ①Yes ② No ③ It depends on the situation statements
3. Do you agree with the statement "Only PLHIV who stopped illegal activities should be given care and treatment "? ①Yes ② No ③ It depends on the situation statements
4. Do you agree with the statement "Doctors should treat PLHIV the same as other patients "? ①Yes ② No ③ It depends on the situation statements
5. Do you agree with the statement "PLHIV should be allowed to work with others "?

①Yes ② No ③ It depends on the situation statements

1. Do you agree with the statement "PLHIV should be allowed to participate in social activities "? ①Yes ② No ③ It depends on the situation statements
2. Do you agree with the statement "PLHIV should be segregated "?

①Yes ② No ③ It depends on the situation statements

1. Do you agree with the statement "PLHIV should be treated the same like other patients "?

①Yes ② No ③ It depends on the situation statements

1. Do you agree with the statement "PLHIV but not yet showing symptoms should be allowed to continue teaching "? ①Yes ② No ③ It depends on the situation statements
2. Do you agree with the statement "PLHIV should be abandoned by his/her family "?

①Yes ② No ③ It depends on the situation statements

1. Do you agree with the statement "I am willing to make friends with PLHIV "? ①Yes ② No ③ It depends on the situation statements
2. Do you agree with the statement "PLHIV would be dispelled by his/her family "?

①Yes ② No ③ It depends on the situation statements

1. Do you agree with the statement "PLHIV would be insulted by his/her classmates "?

①Yes ② No ③ It depends on the situation statements

1. Do you agree with the statement "PLHIV would be stigmatized and discriminated "?

①Yes ② No ③ It depends on the situation statements

1. Do you agree with the statement "PLHIV would be abandoned by his partner or spouse "?

①Yes ② No ③ It depends on the situation statements

**Part IV: Practice and Willingness**

1. Had you experienced sexual intercourse?

①Yes ② No ③ Not sure

1. When did you start to have sex?

①Under the age of 14 years ② 14-18 ③ Older than 18 years

1. How often do you use condoms during sexual intercourse in the past 6 months ?

①Never ② once in a while ③ Sometimes ④ Every time

1. What is your chance of catching HIV?

① No at all ② Not sure ③ Low ④ Moderate ⑤ High

1. Have you ever utilized HCT services? ①Yes ② No
2. Do you know TWFFL ("Together We Fight For Love") to provide free HCT service?"

①Yes ② No ③ Not sure

1. If you were offered free HTC service, would you wish to accept?

①Yes ② No

1. Do you think it is necessary to provide a free HIV test in your university?

①Yes ② No ③ I don't care
